# Automatic Segmentation of Anatomical Structures from CT Scans of Thorax for RTP

**DOI:** 10.1155/2014/472890

**Published:** 2014-12-18

**Authors:** Emin Emrah Özsavaş, Ziya Telatar, Bahar Dirican, Ömer Sağer, Murat Beyzadeoğlu

**Affiliations:** ^1^Electrical and Electronics Engineering Department, Faculty of Engineering, Ankara University, Gölbaşı, 06830 Ankara, Turkey; ^2^Radiation Oncology Department, Gülhane Military Medical Academy, Etlik, 06018 Ankara, Turkey

## Abstract

Modern radiotherapy techniques are vulnerable to delineation inaccuracies owing to the steep dose gradient around the target. In this aspect, accurate contouring comprises an indispensable part of optimal radiation treatment planning (RTP). We suggest a fully automated method to segment the lungs, trachea/main bronchi, and spinal canal accurately from computed tomography (CT) scans of patients with lung cancer to use for RTP. For this purpose, we developed a new algorithm for inclusion of excluded pathological areas into the segmented lungs and a modified version of the fuzzy segmentation by morphological reconstruction for spinal canal segmentation and implemented some image processing algorithms along with them. To assess the accuracy, we performed two comparisons between the automatically obtained results and the results obtained manually by an expert. The average volume overlap ratio values range between 94.30 ± 3.93% and 99.11 ± 0.26% on the two different datasets. We obtained the average symmetric surface distance values between the ranges of 0.28 ± 0.21 mm and 0.89 ± 0.32 mm by using the same datasets. Our method provides favorable results in the segmentation of CT scans of patients with lung cancer and can avoid heavy computational load and might offer expedited segmentation that can be used in RTP.

## 1. Introduction 

Computed tomography (CT) scans are primarily used for diagnostic purposes; however, they may also be used in radiation treatment planning (RTP). Detailed CT scans of patients with cancer are acquired for RTP purposes and used for localizing the tumor and organs at risk (OARs). Optimal RTP requires precise definition of target and critical structures to achieve the best radiotherapeutic outcomes in terms of toxicity and cure. Modern radiotherapy techniques such as intensity modulated radiation therapy (IMRT) are vulnerable to delineation inaccuracies due to the steep dose gradient around the target. In this aspect, accurate contouring is extremely important and comprises an indispensable part of RTP in the modern era.

The delineation procedure is traditionally performed by an expert in radiation oncology with meticulous assessment of CT images of a given patient followed by manual construction of the structure set by drawing two-dimensional contours of every structure in consecutive CT slices. Depending on the tumor site and number of slices to be contoured, delineation of the target and OARs for precise RTP can be quite time-consuming and labor-intensive. Moreover, there is generally no consensus on accurate contouring of target and OARs to guide this critical component of RTP because of the fuzziness of image objects. Contouring procedures performed by different experts may differ substantially and a single expert may even contour the same CT image differently on consequent occasions, referred to as “interobserver variability” and “intraobserver variability,” respectively [1–3]. Discordance, inconsistency, and even slight inaccuracies in the delineation procedure may result in significant variations in target and OAR definition of a given patient, which may have a profound impact on treatment outcomes. Thus, methods to improve consistency, concordance, and accuracy of the contouring procedure for precise RTP are needed.

Automated segmentation algorithms are increasingly used in RTP to optimize the delineation procedure. Robust algorithms significantly expedite the contouring and improve consistency and concordance. The implementation of these sophisticated methods in radiation oncology practice may have implications particularly in busy clinics.

Segmentation of the lungs, trachea/main bronchi, and spinal canal plays a central role in RTP for lung cancer. Most of the lung segmentation approaches [4–6] use thresholding based on the density values followed by connectivity analysis, postprocessing for lung separation, and lung closing to include the high density structures such as vessels and juxtapleural nodules into the segmented lungs. The juxtapleural nodules and pulmonary vessels abutting the medial lung border may inadvertently be excluded from the segmented lungs when lung closing operation is not sufficient. In order to address this undersegmentation issue, a number of approaches have been suggested in the literature [7–17].

de Nunzio et al. [7] applied morphological three-dimensional closing with a fixed size (diameter of 30 mm) spherical structural element to the segmented lungs for inclusion of pleural and internal nodules and to patch the concavities corresponding to vessels.

Yim and Hong [10] proposed a curvature based method for correcting the segmented lung boundary.

Wang et al. [11] developed a texture analysis based method for accurate segmentation of lungs with severe interstitial lung disease.

Sluimer et al. [13] proposed a segmentation-by-registration scheme that is applicable to severe lung pathologies. Major drawbacks of this method include its complexity and the long computation time. Van Rikxoort et al. [14] improved this method by adding an error detection step.

Prasad et al. [15] used the curvature of the ribs in the process of thresholding to segment the lungs in the presence of pulmonary disease. Although the method conferred accurate results in this case, it may fail in the presence of lungs with high density pathologies such as large tumors.

Shape models [16, 17] have also been suggested in lung segmentation; however, these models require a large amount of training data. Furthermore, if a lung shape that cannot be explained by the model is encountered, it needs to be added to the learning set [17].

Airway tree segmentation is critical in correcting the results of lung segmentation, that is, elimination of the external airways from the segmented lungs. Most of the airway tree segmentation methods use region growing and morphological operators applied on the density values [18–21]. Other methods are based on fuzzy connectivity [22], pattern recognition [23], and template matching [24].

Methods for segmenting the spinal cord and the spinal canal include knowledge-based approach [25], atlas-based approach [26], multilevel thresholding, and morphological image processing [27]. Most of them utilize extraction of the vertebra.

Banik et al. [27] performed automatic identification of the rib structure, vertebral column, and spinal canal of pediatric patients. In order to segment the spinal canal, they utilized the Hough transform and the opening-by-reconstruction procedure.

Haas et al. [28] proposed an approach including presegmentation, anatomic orientation, and structure segmentation processes for the automatic segmentation of thoracic and pelvic CT images used for RTP.

In this study, we suggest a fully automated method to segment the lungs, trachea/main bronchi, and spinal canal accurately from CT scans of patients with lung cancer to be used for RTP purposes. Our method consists of three processes. First, the body region of the patient in a CT image is segmented by elimination of the background. Second, rough segmentation of the lung fields, segmentation and elimination of the trachea/main bronchi, the lung fields correction, the right and left lung seperation steps, and a postprocessing step for inclusion of excluded pathological areas into the segmented lungs are realized, respectively. Third, the vertebra and finally the spinal canal are segmented by means of the fuzzy segmentation algorithm. Within these processes, a new algorithm for inclusion of excluded pathological areas into the segmented lungs, a modified version of the fuzzy segmentation by morphological reconstruction for spinal canal segmentation, and the well-known image processing algorithms were used.

Section 2 explains our materials and method in detail.  Section 3 presents the results obtained from our method. Finally, discussion and concluding remarks are given in Section 4.

## 2. Materials and Methods

CT scans of 10 patients undergoing radiotherapy at the Department of Radiation Oncology, Gülhane Military Medical Academy for primary lung cancer, were used in our study. Informed consents of all patients were taken before CT acquisition at the dedicated CT-simulator (GE Lightspeed RT, GE Healthcare, Chalfont St. Giles, UK). Slices are 512 × 512 pixel, 16-bit gray level matrices; and pixel size ranges between 0.76 mm and 1.27 mm. The average number of slices per scan is 100 (range: 79 to 129 slices) while slice thickness ranges between 2.5 mm and 5 mm.

In addition to the 10 CT scans of the 10 patients with lung cancer, we also used 10 different thoracic CT scans from the Lung Image Database Consortium (LIDC) [29] which is a public database headed by the US National Cancer Institute.

To compare our method with other methods, we implemented the algorithms proposed in other studies [7, 10, 11, 27].

**Figure 1:**
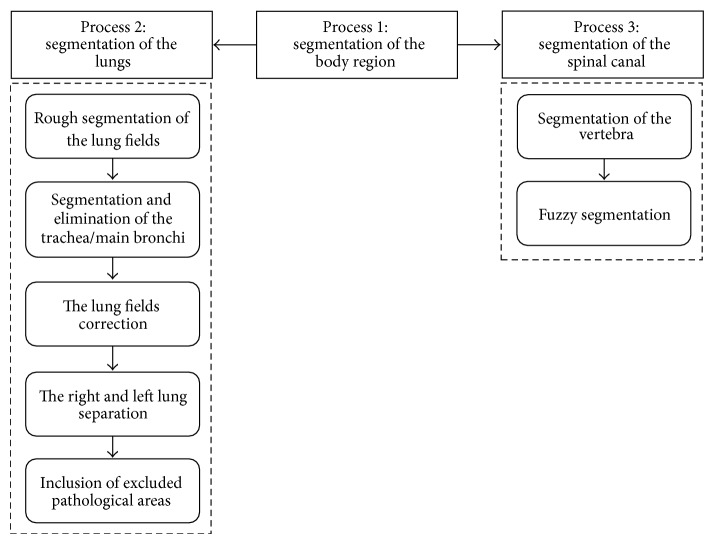
Workflow of the study.

Figure 1 shows the workflow in our study.

### 2.1. Segmentation of the Body Region

**Figure 2:**
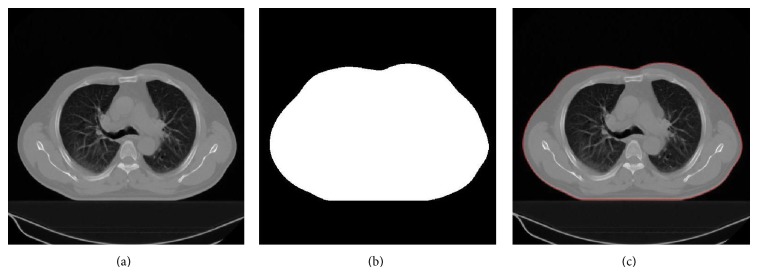
Segmentation of the body region: (a) original CT slice, (b) segmented body region in white, and (c) body contour in red superimposed on the original slice.

The body region is segmented from a CT image by thresholding. First, the Hounsfield Unit-HU (range: −175 to 750) [28] is used as the threshold and then hole-filling procedure is applied to the thresholded results. Segments greater than 800 mm^2^ [28] are accepted as the body region (Figure 2).

### 2.2. Segmentation of the Lungs

**Figure 3:**
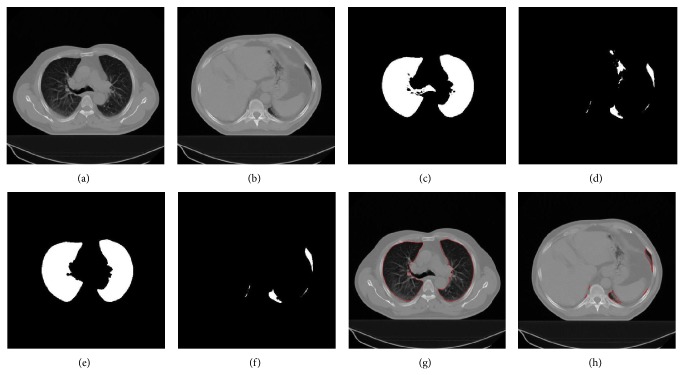
Segmentation of the lungs: (a, b) original CT slices, (c, d) rough segmentation of the lung fields of (a, b) in white, (e) lungs in white after eliminating the bronchi from (c), (f) lungs in white after removing intestine from (d), and (g, h) lung contours in red superimposed on the original slices.

As shown in Figure 3, the main steps of lung segmentation are the rough segmentation of the lung fields, segmentation and elimination of the trachea/main bronchi, and then making the lung fields correction. These steps are followed by the right and left lung separation and inclusion of excluded pathological areas.

#### 2.2.1. Rough Segmentation of the Lung Fields

The body segments of each slice are thresholded using the value of −300 HU [11] and then connected component labelling is applied to the thresholded results.

#### 2.2.2. Segmentation and Elimination of the Trachea/Main Bronchi

A three-dimensional region growing is used to segment the trachea and main bronchi. Within the body segments of the first (upper) few slices, all the pixels with density values lower than −900 HU are labelled as air-filled since air has very low HU values around −1000 in CT slices. By using the connected component analysis, the air-filled region closest to the center of the corresponding body segment with the maximum area is labelled as the trachea.

Using the center pixel of the trachea region as a seed, three-dimensional region growing is applied repeatedly with increasing values of the threshold. Here, initial value of the threshold is −900 HU and we take 64 HU as a value of increment. In order to find the center pixel of the trachea, average location of the pixels in the trachea is calculated.

**Figure 4:**
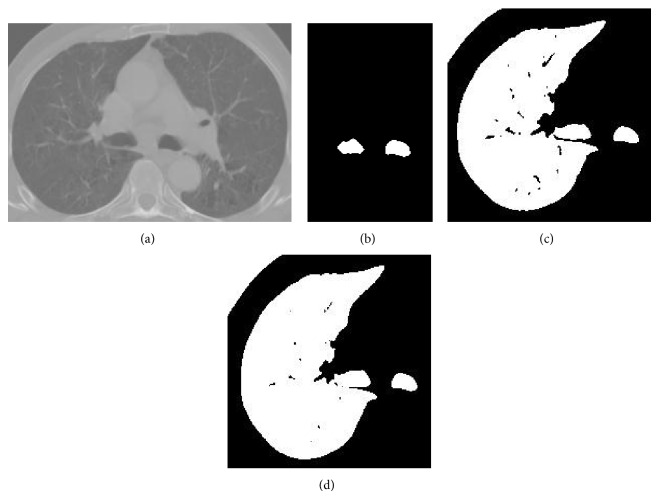
Results of three-dimensional region growing for a CT slice: (a) original CT slice, (b) used threshold which is −836 HU, (c) used threshold which is −772 HU, and (d) used threshold which is −708 HU.

If the segmented structures have a total volume at least twice the structures segmented with the previous threshold, it is considered that the growing region penetrates through the bronchial wall and enters into the lung parenchyma. In this case, value of the increment is reduced by half. This operation is terminated when the increment reaches the value of 1 HU and leakage into the lung field is detected synchronously.  Figure 4 shows the segmented trachea/main bronchi areas using different thresholds.

**Figure 5:**
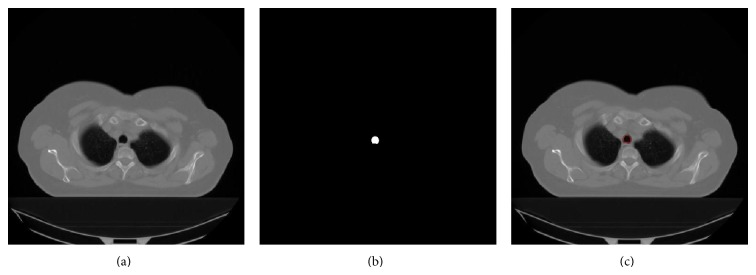
Segmentation of the trachea/main bronchi: (a) original CT slice, (b) segmented trachea/main bronchi in white, and (c) trachea/main bronchi contour in red superimposed on the original slice.

To include the airway wall with higher density values than the air-filled region (lumen), we apply morphological dilation with a 3 × 3 disk-shaped structuring element [30]. Segmented structures are labelled as the trachea/main bronchi as shown in Figure 5 and removed from the rough lung fields. Here, the morphological dilation alters the borders in a way that the trachea/main bronchi include the airway wall as intended.

We fill the holes stemming from vessels, nodules, tumors, or other high density pathologies that are inside the lung fields using a hole-filling algorithm. Finally, morphological closing with a 3 × 3 disk-shaped structuring element [30] is applied twice to the lungs to obtain the pulmonary borders clearly.

#### 2.2.3. The Lung Fields Correction

A three-dimensional evaluation of the CT scan is performed to remove intestine that has similar density values as the lungs. Intestine appears in the lower (caudal) slices of the scan. All the rough lung regions smaller than 200 mm^2^ are eliminated and connectivity is checked within the remaining lung regions by means of three-dimensional region growing [7], using the pixels that belong to rough lung regions in the central slice of the scan as the seeds.

#### 2.2.4. The Right and Left Lung Separation

If a slice contains a lung region wider than half of the width of the body region, then separation of the connected lungs is performed by identifying the anterior and posterior junctions as follows.

**Step 1 .**

Find the bounding box (BB) of the lung region and determine the boundaries of BB as BB.left, BB.right, BB.top, and BB.bottom.

**Step 2 .**

In order to generate the anterior and posterior junction lines, determine a region of interest (ROI) using the border definitions below:

(1) $$\begin{array}{c} \text{ROI}.\text{left}=\text{BB}.\text{left}+\frac{\left(\text{BB}.\text{right}-\text{BB}.\text{left}\right)}{3},\\ \text{ROI}.\text{right}=\text{BB}.\text{left}+2\left(\frac{\left(\text{BB}.\text{right}-\text{BB}.\text{left}\right)}{3}\right),\\ \text{ROI}.\text{top}=\text{BB}.\text{top},\\ \text{ROI}.\text{bottom}=\frac{\left(\text{BB}.\text{top}+\text{BB}.\text{bottom}\right)}{2}. \end{array}$$

**Step 3 .**

Find the greatest nonlung component that is in the middle upper part of the ROI.

**Step 4 .**

For anterior junction line, find the pixel of the nonlung component obtained from the previous step with minimum row position, that is, nearest pixel to the ROI.top and closest to the center column of the ROI. Save this pixel as *P*(*r*, *c*), where *r* and *c* show the row and column position of this pixel, respectively.

**Step 5 .**

Compare the density values of *P*(*r* − 1, *c* − 1), *P*(*r* − 1, *c*), and *P*(*r* − 1, *c* + 1). Mark the pixel with maximum density value as the new *P*(*r*, *c*).

**Step 6 .**

If *P*(*r*, *c*) belongs to the lung region, label it as nonlung and go to Step 5.

**Step 7 .**

For posterior junction line, find the pixel of the nonlung component obtained from Step 3 with maximum row position, that is, nearest pixel to the ROI.bottom and closest to the center column of the ROI. Save this pixel as *P*(*r*, *c*), where *r* and *c* show the row and column position of this pixel, respectively.

**Step 8 .**

Compare the density values of *P*(*r* + 1, *c* − 1), *P*(*r* + 1, *c*), and *P*(*r* + 1, *c* + 1). Mark the pixel with maximum density value as the new *P*(*r*, *c*).

**Step 9 .**

If *P*(*r*, *c*) belongs to the lung region, label it as nonlung and go to Step 8. Otherwise, terminate the processing.

**Figure 6:**
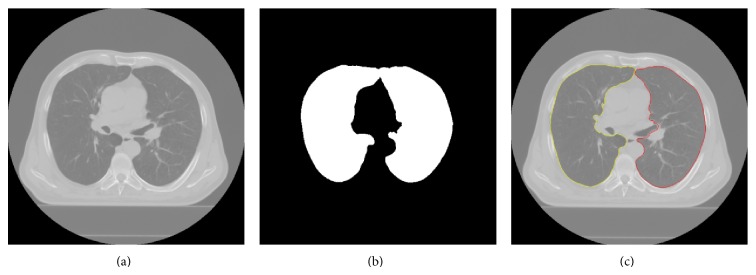
The right and left lung separation: (a) original CT slice, (b) connected lungs in white, and (c) right lung contour in yellow and left lung contour in red superimposed on the original slice.

The result is shown in Figure 6.

#### 2.2.5. Inclusion of Excluded Pathological Areas

Although the lung segmentation based on thresholding is simple and quite fast, it may fail in case of lungs with large tumors of high density since a significant contrast between the lungs and the surrounding tissues does not exist. In such circumstances, morphological operations like closing may not be sufficient to correct the borders of the lungs.

We propose a three-stage approach to include pathological areas, that is, tumors that are in relation with the borders of the lungs and are excluded by segmentation in the previous steps, into the lungs. This approach is based on obtaining the intersection of interpolated and propagated lungs.

**Stage 1 .**

Apply an interpolation procedure to the lungs obtained from the previous subprocess.

**Step 1 .**

Create an empty mask, that is, a two-dimensional matrix the same size as the slices.

**Step 2 .**

Starting from the first (upper) slice to the last (lower) slice of CT scan, if a pixel *P*(*x*, *y*) is labelled as lung in any slice, label the corresponding pixel *P*(*x*, *y*) in the mask as lung.

**Step 3 .**

For each pixel *P*(*x*, *y*) labelled as lung in the mask, find the first (*f*) and the last (*l*) slices of CT scan in which *P*(*x*, *y*) is labelled as lung.

**Step 4 .**

Label all the pixels *P*(*x*, *y*) as lung in all of the slices between *f* and *l*, where *x* and *y* are the coordinates of the pixel *P*.

**Stage 2 .**

Propagate the right and left lungs obtained from the previous subprocess separately.

**Step 1 .**

Find the border pixels of the lung.

**Step 2 .**

For each border pixel, find the nonlung pixels within the neighborhood (7 × 7) centered at the current border pixel and label them as candidate pixels.

**Step 3 .**

For each candidate pixel, if more than half of the pixels within the neighborhood (7 × 7 × 7) centered at the current candidate pixel are labelled as lung, schedule the current candidate pixel for inclusion into the lung.

**Step 4 .**

If there is any scheduled candidate pixel, label them as lung and go to Step 1. Otherwise terminate the processing.

**Stage 3 .**

Get the intersection of the lungs that resulted from Stages 1 and 2.

**Figure 7:**
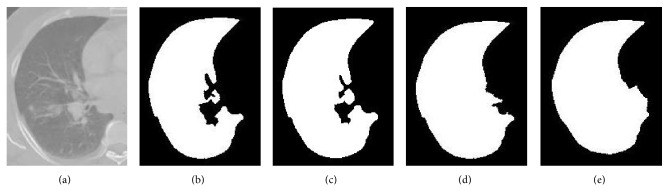
Effects of different neighborhoods used in Section 2.2.5, Stage 2, Step 3: (a) original CT slice, (b) segmented right lung, (c) propagated right lung using 5 × 5 × 5 neighborhood, (d) propagated right lung using 7 × 7 × 7 neighborhood, and (e) propagated right lung using 9 × 9 × 9 neighborhood. Using 7 × 7 × 7 neighborhood, excluded pathological areas are included into the lungs successfully.

**Figure 8:**
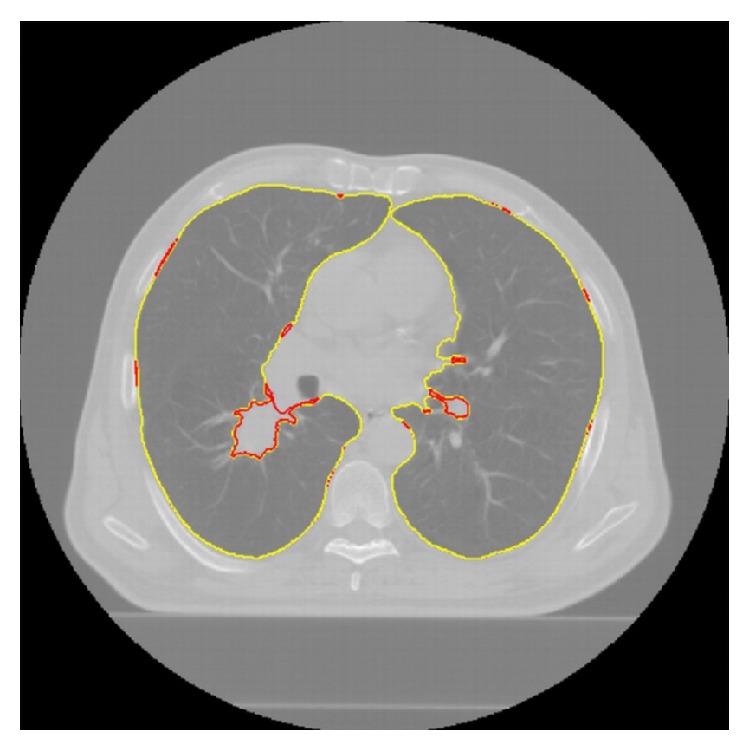
Inclusion of excluded pathological areas: pathological areas contours in red superimposed on the original slice.

Herein, neighborhood of 7 × 7 × 7 region was chosen experimentally by testing this approach on the CT scans from the Department of Radiation Oncology, Gülhane Military Medical Academy. These CT scans belong to 10 patients with limited-stage small cell lung cancer. Each of the patients has one tumor. Gross tumor volumes (GTVs) are 36.7, 16.4, 85.8, 33.0, 28.7, 40.8, 66.5, 21.7, 45.5, and 93.2 cc.  Figure 7 shows the effects of different neighborhoods used in Stage 2, Step 3. After all, as shown in Figure 8, excluded pathological areas are included into the lungs.

In order to see results of this approach in case of juxtapleural nodules, we used 10 CT scans from the LIDC. The CT scans from the LIDC do not include large tumors but comprised a total of 12 juxtapleural nodules that are 5.0–10.0 mm in diameter.

As can be seen from Section 3, the lungs can be segmented accurately in both cases using this approach.

### 2.3. Segmentation of the Spinal Canal

Fuzzy segmentation approach is examined in segmentation of the spinal canal. This process includes two subprocesses: segmentation of the vertebra and fuzzy segmentation of the spinal canal.

#### 2.3.1. Segmentation of the Vertebra

Since the spinal canal is the space in vertebra through which the spinal cord passes, vertebra must be segmented initially. Bones have higher density values than other structures so that the body region is thresholded with a value of 145 HU [28] in each slice. Using the connected component labelling, the regions that are greater than 25 mm^2^ and have at least a distance of 10 pixels to the border of the body segments are accepted as bone segments. Exploiting the advantage of anatomic knowledge, a region of interest is determined and vertebra is segmented as follows.

**Step 1 .**

Find the bounding box (BB) of the body segment and determine the boundaries of BB as BB.left, BB.right, BB.top, and BB.bottom.

**Step 2 .**

Determine the coordinates of the center point (CP) of BB as CP.row and CP.col.

**Step 3 .**

Determine a region of interest (ROI) for vertebra detection using the border definitions below:

(2) $$\begin{array}{c} \text{ROI}.\text{left}=\text{CP}.\text{col}-10,\\ \text{ROI}.\text{right}=\text{CP}.\text{col}+10,\\ \text{ROI}.\text{top}=\text{CP}.\text{row}-50,\\ \text{ROI}.\text{bottom}=\text{BB}.\text{bottom}-10. \end{array}$$

**Step 4 .**

Label the bone segment that overlaps with the ROI as vertebra and create a binarized vertebra image for the corresponding slice.

**Step 5 .**

In the vertebra image detect the pixels that have nonzero gradient magnitude values and label them as vertebra in order to include the missing parts.

#### 2.3.2. Fuzzy Segmentation

Banik et al. [27] used morphological reconstruction to segment the spinal canal instead of fuzzy connectivity. They applied the Hough transform to detect the best fitting circles to the cropped edge maps that include the thoracic vertebral structure and then used the centers of these circles as the seeds for the morphological reconstruction.

We use a modified version of the fuzzy segmentation by morphological reconstruction presented in aforementioned study [27]. The proposed spinal canal segmentation method detects the seeds and performs fuzzy segmentation only for slices in which the spinal canal is not enclosed completely by the vertebra.

The steps of the fuzzy segmentation approach that we propose are as follows.

**Step 1 .**

Determine if the slice has a spinal canal enclosed completely by the vertebra or not. To do this, find the bounding box of the vertebra. In the bounding box of the vertebra, find the pixels that are not labelled as bone. Using connected component labelling, detect the segments that the pixels form. If the slice has a segment that is not connected to the boundaries of bounding box of the vertebra, it is understood that the slice has a spinal canal enclosed completely by the vertebra.

**Step 2 .**

If so, label the nonbone region enclosed by the vertebra as the spinal canal and then go to Step 9.

**Step 3 .**

If not, take CP of the spinal canal in the previous (following) slice as a seed.

**Step 4 .**

Take a window of size 11 × 11 pixels centered at the coordinates of CP in the current slice. Detect the pixels in the defined window having density values in the range of *μ* ± 2.5*σ*, where *μ* = 23 HU and *σ* = 15 HU. Calculate the mean *μ*_SP_ and the standard deviation *σ*_SP_ of the density values of the detected pixels.

**Step 5 .**

Reconstruct the fuzzy region according to the fuzzy membership function, namely, the unnormalized Gaussian function, using *μ*_SP_ and *σ*_SP_ as the mean and the standard deviation parameters and the pixels detected in the previous step as the seeds.

**Step 6 .**

Binarize the fuzzy region using 0.5 as the threshold value.

**Step 7 .**

Using the connected component labelling, find the segment with the maximum area in the thresholded fuzzy region and label it as the spinal canal.

**Step 8 .**

Morphologically open [30] the spinal canal with a 3 × 3 disk-shaped structuring element to obtain the border regions clearly.

**Step 9 .**

Take the following (previous) slice and go to Step 1.

**Figure 9:**
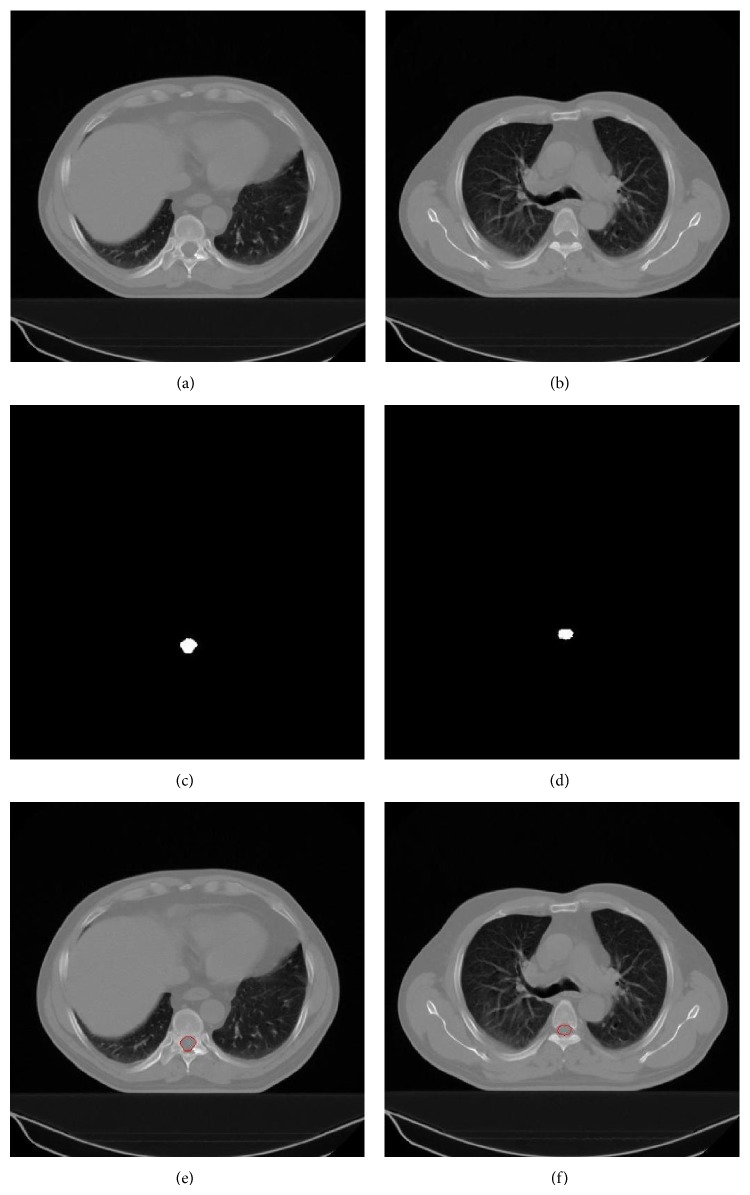
Segmentation of the spinal canal: (a) original CT slice in which the vertebra encloses the spinal canal completely, (b) original CT slice in which the vertebra does not enclose the spinal canal completely, (c, d) segmented spinal canals of (a, b) in white, and (e, f) spinal canal contours in red superimposed on the original slices.

The result of this process is shown in Figure 9.

## 3. Results and Discussion

**Figure 10:**
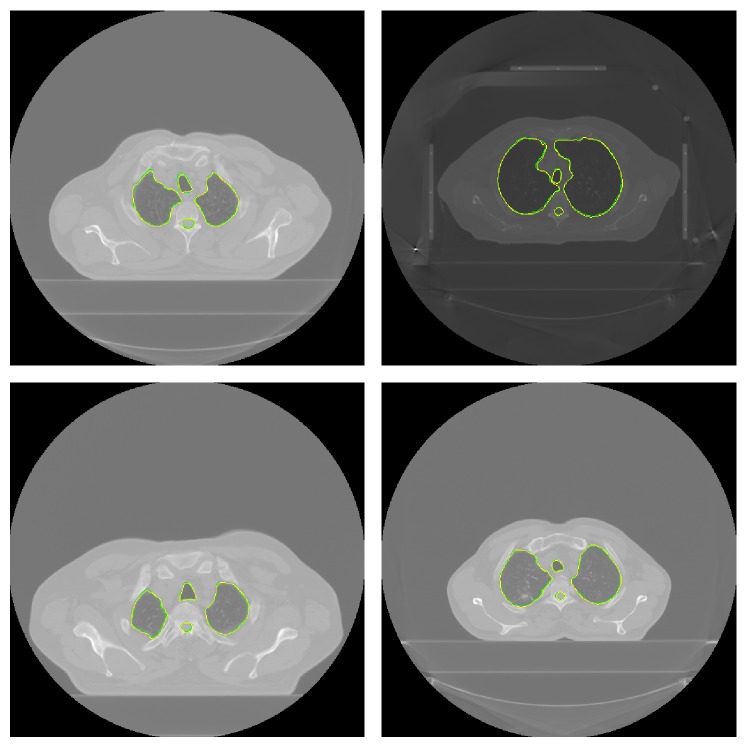
Automatic and manual segmentation of the lungs, trachea/main bronchi, and spinal canal: contours in yellow show automatic segmentation results and contours in green show manual segmentation results.

**Figure 11:**
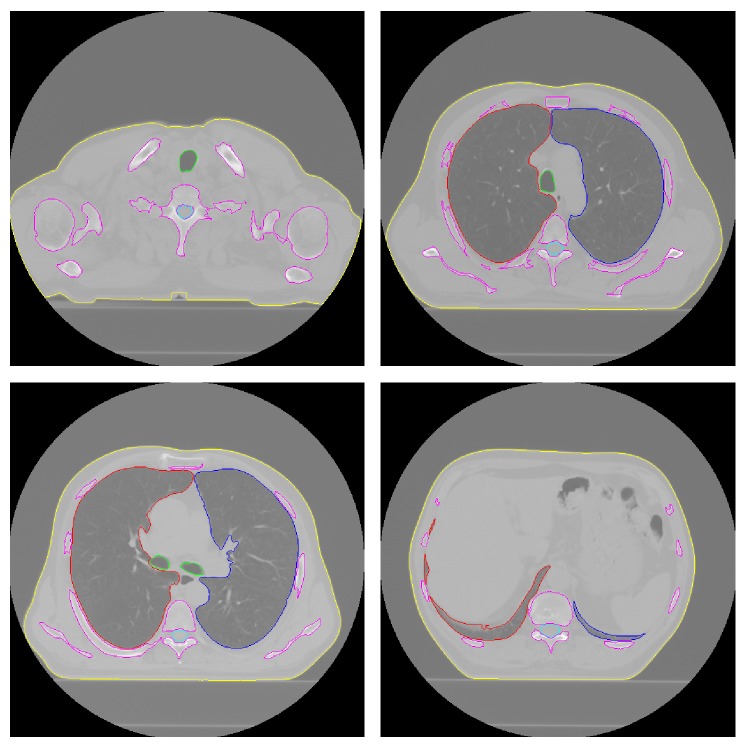
The contours of automatically segmented structures: body contours in yellow, left lung contours in blue, right lung contours in red, trachea/main bronchi contours in green, bone contours in magenta, and spinal canal contours in cyan superimposed on the original slices.

The proposed segmentation method was implemented in Matlab R14 and tested on a PC with 1.73 GHz processor and 1.0 GB RAM. Also, we implemented the methods proposed in other studies [7, 10, 11, 27] as described in Introduction and applied them along with our method to the 10 CT scans of patients with cancer and to the 10 CT scans from the LIDC on the same platform mentioned above. Figures 10 and 11 show results of our method for randomly selected slices from the 10 CT scans of patients with cancer.

Similar to other studies [7, 10, 11], to segment the lungs and trachea/main bronchi, we used combinations of well-known image processing algorithms, namely, thresholding, morphological operations, region growing, and connected component labelling.

In order to solve the undersegmentation problem caused by pleural nodules and pulmonary vessels contacting the lung boundary, de Nunzio et al. [7] applied morphological three-dimensional closing to the segmented lungs, and Yim and Hong [10] corrected the lung boundaries in each slice by evaluating curvatures of the boundary pixels. Wang et al. [11] created texture feature images using a cooccurrence matrix and thresholded multiplication of entropy and inverse difference moment feature values of each pixel to identify the missed abnormal lung regions in CT slices.

In this study, we propose an original method to obtain pathological areas in the segmented lungs as described in Section 2.2.5, based on obtaining the intersection of the interpolated and propagated lungs.

Banik et al. [27] utilized a fuzzy segmentation approach by using morphological reconstruction to segment the spinal canal. For each slice they applied the Hough transform to detect the best fitting circles to the cropped edge maps that include the thoracic vertebral structure and then used the centers of these circles as the seeds for the morphological reconstruction. Our approach to segment the spinal canal differs from the method proposed by Banik et al. [27] in two ways. First, unlike the method proposed by Banik et al. [27], we detect the seeds and perform fuzzy segmentation only for slices in which the spinal canal is not enclosed completely by the vertebra. We segment the nonbone region enclosed by the vertebra as the spinal canal if it is enclosed completely by the vertebra. Second, we take simply the center point of the segmented spinal canal in the previous (following) slice as a seed without requiring any time-consuming process like the Hough transform.

To assess the accuracy, we performed two comparisons between the automatically obtained results and the gold standard. Here, the results obtained manually by an expert were used as gold standard. An expert radiation oncologist manually delineated the body region, right and left lung, trachea/main bronchi, and spinal canal in consecutive slices of all CT scans by mouse dragging at a dedicated contouring workstation using Advantage SimMD simulation and localization software (Advantage SimMD, GE, UK) [31, 32].

The first comparison was performed by computing the volume overlap ratio (VOR) [4, 33], that is, ratio of the intersection volume to the union volume, as follows:

(3) $$\begin{array}{c} \text{VOR}=\frac{V\left(A\cap M\right)}{V\left(A\cup M\right)}\times 100, \end{array}$$

where *A* and *M* are automatically and manually segmented structures, respectively.

Volume of a structure is computed by taking the product of total number of pixels labelled as that structure, pixel dimensions (width and height), and slice thickness.

As the second comparison, surface distance evaluation [33] was performed to account for the global and local disagreement between automatically and manually segmented structures precisely. Herein the average, root mean square (RMS), and maximum symmetric surface distance values between the surface voxels of manually and automatically segmented structures were assessed. To that end, the voxels in a segmented structure having at least one neighbor (within 18 neighborhoods) that does not belong to the structure are defined as the surface voxels. After defining the sets of surface voxels for manually and automatically segmented structures, for each surface voxel in these sets, the Euclidean distance to the closest surface voxel in the other set is calculated.

For a given structure, the average symmetric surface distance (ASD), RMS symmetric surface distance (RMSD), and maximum symmetric surface distance (MSD) are calculated using the following equations:

(4) $$\begin{array}{c} \text{ASD}=\frac{\sum_{V_{A}\in S_{A}}d\left(V_{A},S_{M}\right)+\sum_{V_{M}\in S_{M}}d\left(V_{M},S_{A}\right)}{\left|S_{A}\right|+\left|S_{M}\right|},\\ \text{RMSD}=\sqrt{\frac{\sum_{V_{A}\in S_{A}}d^{2}\left(V_{A},S_{M}\right)+\sum_{V_{M}\in S_{M}}d^{2}\left(V_{M},S_{A}\right)}{\left|S_{A}\right|+\left|S_{M}\right|}},\\ \text{MSD}=\max\left\{\underset{V_{A}\in S_{A}}{\max}\left\{d\left(V_{A},S_{M}\right)\right\},\underset{V_{M}\in S_{M}}{\max}\left\{d\left(V_{M},S_{A}\right)\right\}\right\}, \end{array}$$

where *S*_*A*_ and *S*_*M*_ represent the sets of surface voxels of automatically and manually segmented structures, respectively, while *d*(*v*, *S*) refers to the minimum Euclidean distance between a voxel *v* and surface *S*, and |*S*| is the number of voxels that belong to the surface *S*.

Comparison of the results of our method and the other methods with the gold standard is shown in Tables 1, 2, 3, and 4 for the 10 CT scans from the LIDC and in Tables 5, 6, 7, and 8 for the 10 CT scans of the 10 cancer patients.

To make a performance assessment of the proposed segmentation method, we compared the average processing time measured from all scans for segmentation of the lungs, trachea/main bronchi, and spinal canal with the methods proposed in other studies [7, 10, 11, 27]. For this purpose, by means of “tic” and “toc” commands of Matlab, we started a stopwatch timer and display the elapsed time to segment a structure in a slice using one of the methods. We obtained the elapsed time values for each method, each structure, and each slice separately. After then, for each method, the mean of the time values to segment a structure in a slice is calculated and multiplied by 100.  Table 9 shows average processing time of the methods for a 512 × 512 × 100 CT scan.

## 4. Conclusions

In this study, we suggest a fully automated method to segment the lungs, trachea/main bronchi, and spinal canal from CT scans of thorax intended for use in RTP. For this purpose, we implemented software that performs three processes. In the first process, the body region of the patient was segmented by elimination of the background. The lungs and trachea/main bronchi were segmented in the second process and finally, the spinal canal was segmented. Within these processes, a new algorithm for inclusion of excluded pathological areas into the segmented lungs, a modified version of the fuzzy segmentation by morphological reconstruction for spinal canal segmentation, and the well-known image processing algorithms were used. The fixed threshold values and the size of the structuring elements we utilized were determined on the basis of our experiments and the other studies in the literature.

Comparison of our method with the gold standard using the LIDC data reveals that the proposed method properly reproduces the manual segmentations, similar to other methods (Tables 1–4). Furthermore, our method provides better results in the segmentation of CT scans of patients with lung cancer (Tables 5–8). We obtained lower distance values than the methods proposed in [7, 10, 11]. This confirms that our method ensures better agreement with the gold standard. Our superior results in lung segmentation may be explained with the inclusion of an additional subprocess, namely, “inclusion of excluded pathological areas” which is unique to our study.

As shown in Table 9, our lung segmentation method is 1.3, 1.7, and 2.3 times faster than the methods proposed in other studies [7, 10, 11], respectively. This is because, unlike the methods proposed in other studies [7, 10, 11], our method does not utilize time-consuming processes, that is, morphological three-dimensional closing, scan line search, and texture feature calculation. While the number of required calculations in our lung segmentation method is 9, the methods proposed in those studies [7, 10, 11] require 9, 10, and 12 calculations, respectively.

Our modified version of the fuzzy segmentation by morphological reconstruction has achieved comparable results to the one presented in the aforementioned study [27], while decreasing the computational load and speeding up the process of spinal canal segmentation. On average, our spinal canal segmentation process takes 4.4 minutes, while the time needed for the method proposed in [27] is 9.3 minutes (Table 9). This may be explained by our different approach in detecting the seeds and omitting fuzzy segmentation in slices where the vertebra encloses the spinal canal completely.

The use of our method in RTP may have potential implications. It may improve consistency and concordance in delineation, which is a critical part of RTP. It may also assist in accelerating the clinical workflow through shortening the time of contouring, which is highly desirable in busy clinics.

In conclusion, our proposed method achieved favorable results in patients with lung cancer. This very concise and effective method can avoid heavy computational load and might offer expedited segmentation that can be used in RTP, despite the need for further studies supporting its utilization.

## Figures and Tables

**Table 1:** Comparison of VOR (%) for the 10 CT scans from the LIDC.

OARs	Method				
	Our method	de Nunzio et al. [7]	Yim and Hong [10]	Wang et al. [11]	Banik et al. [27]
Left lung	99.14 ± 0.33	99.12 ± 0.38	—	—	—
Right lung	99.07 ± 0.30	99.03 ± 0.42	—	—	—
Lungs (together)	99.11 ± 0.26	—	99.15 ± 0.30	98.90 ± 0.30	—
Trachea/main bronchi	96.91 ± 1.47	97.06 ± 1.30	97.68 ± 1.42	95.61 ± 1.65	—
Spinal canal	97.19 ± 2.72	—	—	—	97.11 ± 2.35

**Table 2:** Comparison of the average symmetric surface distance (mm) for the 10 CT scans from the LIDC.

OARs	Method				
	Our method	de Nunzio et al. [7]	Yim and Hong [10]	Wang et al. [11]	Banik et al. [27]
Left lung	0.31 ± 0.10	0.44 ± 0.19	—	—	—
Right lung	0.27 ± 0.17	0.34 ± 0.12	—	—	—
Lungs (together)	0.29 ± 0.03	—	0.32 ± 0.11	0.34 ± 0.20	—
Trachea/main bronchi	0.34 ± 0.14	0.36 ± 0.05	0.39 ± 0.13	0.59 ± 0.15	—
Spinal canal	0.28 ± 0.21	—	—	—	0.35 ± 0.09

**Table 3:** Comparison of the RMS symmetric surface distance (mm) for the 10 CT scans from the LIDC.

OARs	Method				
	Our method	de Nunzio et al. [7]	Yim and Hong [10]	Wang et al. [11]	Banik et al. [27]
Left lung	0.61 ± 0.22	0.79 ± 0.23	—	—	—
Right lung	0.63 ± 0.38	0.82 ± 0.51	—	—	—
Lungs (together)	0.67 ± 0.18	—	0.71 ± 0.22	0.89 ± 0.37	—
Trachea/main bronchi	0.66 ± 0.35	0.69 ± 0.20	0.81 ± 0.24	1.13 ± 0.41	—
Spinal canal	0.60 ± 0.43	—	—	—	0.73 ± 0.38

**Table 4:** Comparison of the maximum symmetric surface distance (mm) for the 10 CT scans from the LIDC.

OARs	Method				
	Our method	de Nunzio et al. [7]	Yim and Hong [10]	Wang et al. [11]	Banik et al. [27]
Left lung	1.76 ± 0.66	1.83 ± 0.99	—	—	—
Right lung	1.93 ± 1.03	2.02 ± 0.87	—	—	—
Lungs (together)	2.08 ± 1.15	—	2.23 ± 1.36	2.76 ± 1.90	—
Trachea/main bronchi	2.55 ± 1.44	2.88 ± 0.97	3.03 ± 1.65	3.51 ± 2.24	—
Spinal canal	2.67 ± 1.89	—	—	—	3.78 ± 2.00

**Table 5:** Comparison of VOR (%) for the 10 CT scans of the 10 cancer patients.

OARs	Method				
	Our method	de Nunzio et al. [7]	Yim and Hong [10]	Wang et al. [11]	Banik et al. [27]
Left lung	98.70 ± 1.32	96.50 ± 0.91	—	—	—
Right lung	98.70 ± 0.86	96.30 ± 1.12	—	—	—
Lungs (together)	98.70 ± 1.27	—	97.10 ± 1.02	95.40 ± 1.82	—
Trachea/main bronchi	94.30 ± 3.93	94.60 ± 3.35	94.60 ± 2.87	93.00 ± 3.63	—
Spinal canal	96.50 ± 3.67	—	—	—	96.70 ± 3.59

**Table 6:** Comparison of the average symmetric surface distance (mm) for the 10 CT scans of the 10 cancer patients.

OARs	Method				
	Our method	de Nunzio et al. [7]	Yim and Hong [10]	Wang et al. [11]	Banik et al. [27]
Left lung	0.73 ± 0.36	0.90 ± 0.51	—	—	—
Right lung	0.77 ± 0.48	0.93 ± 0.71	—	—	—
Lungs (together)	0.63 ± 0.32	—	0.94 ± 0.57	0.99 ± 0.73	—
Trachea/main bronchi	0.89 ± 0.72	0.50 ± 0.23	0.55 ± 0.31	0.71 ± 0.22	—
Spinal canal	0.57 ± 0.41	—	—	—	0.52 ± 0.39

**Table 7:** Comparison of the RMS symmetric surface distance (mm) for the 10 CT scans of the 10 cancer patients.

OARs	Method				
	Our method	de Nunzio et al. [7]	Yim and Hong [10]	Wang et al. [11]	Banik et al. [27]
Left lung	1.33 ± 0.48	1.77 ± 0.63	—	—	—
Right lung	1.48 ± 0.75	1.51 ± 0.89	—	—	—
Lungs (together)	1.30 ± 0.93	—	1.92 ± 1.13	2.16 ± 1.24	—
Trachea/main bronchi	1.52 ± 0.86	1.23 ± 0.55	1.38 ± 0.77	1.56 ± 0.97	—
Spinal canal	1.13 ± 0.78	—	—	—	1.02 ± 0.62

**Table 8:** Comparison of the maximum symmetric surface distance (mm) for the 10 CT scans of the 10 cancer patients.

OARs	Method				
	Our method	de Nunzio et al. [7]	Yim and Hong [10]	Wang et al. [11]	Banik et al. [27]
Left lung	8.42 ± 3.48	9.16 ± 3.01	—	—	—
Right lung	8.23 ± 4.12	10.02 ± 4.65	—	—	—
Lungs (together)	8.57 ± 2.88	—	10.67 ± 2.78	11.13 ± 4.34	—
Trachea/main bronchi	11.78 ± 4.35	11.21 ± 3.98	11.28 ± 4.74	12.96 ± 4.02	—
Spinal canal	8.46 ± 3.97	—	—	—	8.38 ± 5.79

**Table 9:** Average processing time (minutes) of the methods for a 512 × 512 × 100 CT scan.

OARs	Method				
	Our method	de Nunzio et al. [7]	Yim and Hong [10]	Wang et al. [11]	Banik et al. [27]
Lungs	3.8	5.0	6.3	8.9	—
Trachea/main bronchi	1.5	1.8	1.6	1.5	—
Spinal canal	4.4	—	—	—	9.3
